# A study of safety acceptance and behavioral interventions for autonomous driving technologies

**DOI:** 10.1038/s41598-022-22720-0

**Published:** 2022-10-25

**Authors:** Mingyang Deng, Yingshi Guo

**Affiliations:** 1 School of Automobile, Chang’an University, Xi’an, 710064 Shaanxi China; 2 College of Automobile Engineering, Changchun University of Technology College of Humanities and Information, Changchun, 130122 Jilin China

**Keywords:** Mechanical engineering, Human behaviour

## Abstract

Explaining the phenomenon of declining acceptance of automated driving technology (ADT) and predicting trends in acceptance has become an important area of research. To explore the reasons for the decline in acceptance of automated vehicles and how to improve user acceptance, we studied mechanisms of the influence process from the relationship between safety riskiness of ADT and user acceptance, and examined the mediating and moderating effects of the proposed intervention behaviors on the influence relationship between these two. First, an improved acceptance model incorporating safety risk factors was developed. Subsequently, the psychological change process of user acceptance was analyzed based on people’s response to accident information. Ultimately, the results show that safety cognition risk regarding ADT has a significant negative impact on user acceptance. Next, the mediating model where user experience was introduced as a moderating variable was designed. From the test results of this model, it is found that the proposed behavioral intervention strategy is effective in attenuating the degree of impact of the safety riskiness of ADT on acceptance. The risk-based acceptance explanation model and intervention method designed in this study provide a scientific basis and practical approach to develop the market for automated vehicles.

## Introduction

ADT is the core technology for the development of smart cars, and each of its upgraded products has attracted extensive attention from society^1^. User acceptance serves as an effective tool to measure how well the technology is accepted by users and can effectively predict the potential market size and population characteristics of new products. However, with the dynamic changes in the external environment, how to explain the changing trend of public acceptance has become an important research direction in the automotive field^2^. Recently, automated vehicles (AVs) have been involved in a number of traffic accidents, and the user acceptance data have shown a continuous decline since then. In response, scholars have proposed hypotheses to verify the intrinsic link between traffic accidents and declining acceptance^3^. In recent studies, it has been found that more and more users are concerned about the safety and stability of AVs^4,5^.

The first global regulation to certify AVs is the European ECE R157, which was introduced in 2021. Currently, AVs on the market are mainly level 3 automated driving functions. AVs with level 3 are capable of performing all dynamic driving tasks under the system’s design operating conditions, while the human driver is not required to operate it. However, the human driver is required to monitor the driving scenario and be ready to take over the vehicle at any time^6^. In the process of AVs moving from advanced assisted driving to higher-level automated driving, users’ safety cognition and acceptance of technology have become the main factors limiting the commercialization of higher-level automated driving. Due to the users’ misunderstanding about automated driving functions and their applicable conditions, the incorrect use and operation of related functions is the main reason for traffic accidents, and the occurrence of traffic accidents affects the public’s acceptance of ADT^7^. Therefore, this study focuses on the user acceptance study of AVs equipped with level 3 ADT.

In the automotive field, the theories of planned behavior and the technology acceptance model are two main theories that are generally accepted and widely used. Theory of planned behavior (TPB) is the dominant theory for studying the influence of users’ behavioral attitudes on actual actions. Shalender et al.^8^ found that TPB can explain, predict and intervene in the occurrence of a phenomenon through the relationship between psychological variables and actual behavior. Besides that, the Technology Acceptance Model (TAM) was a commonly used model to study the process of external variables influencing the change in users’ technology perceptions and attitudes^9^. This model analyzed the process of changes in users’ psychological activities such as cognition, perception, judgment and decision making by describing the influence of measurable external variables on users’ internal potential variables. However, these two psychological research models have different focuses, with the Theory of Planned Behavior focusing on the process of psychological activity changes that influence the occurrence of behavior, and the TAM focusing on the study of the impact of external information on users’ perceptions. In general, the common feature of these models is the analysis of the intrinsic paradigm in the process of psychological and behavioral changes of users influenced by external factors, which is the main theoretical approach to the study of users’ negative attitudes toward ADT.

In 1960, Bauer^10^ introduced perceived risk into the field of psychological research by presenting it as a negatively correlated factor in the model, making the study of user psychology more objective. In 1983, Derbaix^11^ introduced users’ perceived uncertainty into the concept of perceived risk, which promoted the process of scholars’ research on perceived risk. On this basis, acceptance theory has achieved more achievements through the continuous development of scholars. In recent years, a study by Sun et al.^12^ verified that functional perceived risk was more likely to influence users’ attitudes toward new products than affective perceived risk. Detjen et al.^13^ proposed that perceived risk can influence people around through subjective norm and both had significant effects. The Hegner et al.^14^ found that as higher levels of automated driving features were developed, more and more vehicles were equipped with different levels of automated driving systems, and found that people were overly dependent on them. As a result, driving interference and improper operation become the main causes of automated vehicle accidents. In addition, a study by Liu et al.^15^ concluded that since the current marketed ADT cannot replace the driver to cope with the complex road environment, the misalignment of different levels of technology and products has become the main cause of recent traffic accidents.

Statistics from annual traffic accident report have identified an upward trend in the percentage of recent traffic accidents involving vehicles with Level 2 ADT. The increase in automated vehicle accidents may lead to a change in public opinions and attitudes toward ADT. For instance, the studies by Kwon et al.^16^ and Badue et al.^17^ have shown that traffic accidents can create public misunderstanding and antipathy toward ADT, and that low-level product features mislead people to misperceive higher-level technology. Therefore, it is necessary to analyze the impact of potential safety riskiness of AVs on user acceptance in order to find the real reasons for the decline in user acceptance and to provide effective methodological guidance for improving acceptance.

Based on the above analysis, the predicted reasoning process is that after users perceive the safety riskiness of AVs, their positive attitudes toward using ADT products begin to falter, eventually leading to changes in user acceptance. Therefore, we propose a hypothesis that the potential safety riskiness of AVs has a negative correlation effect on the acceptance of ADT. A new model was constructed by using the theory of planned behavior in conjunction with a technology acceptance model, adding external factors characterizing the safety riskiness of AVs and a potential variable portraying users’ perceptions of safety riskiness. Theoretical data analysis was then used to test the research hypotheses and explained the reasons for the decline in acceptance. In addition, the mediating and moderating effects of the designed mediation model were tested according to the classification data of educational degree and driving age in order to analyze the effects of the behavioral intervention. It is concluded that the proposed behavioral intervention approach is more effective in improving users’ re-recognizing on the safety of ADT.

## Construction of the improved model

**Figure 1:**
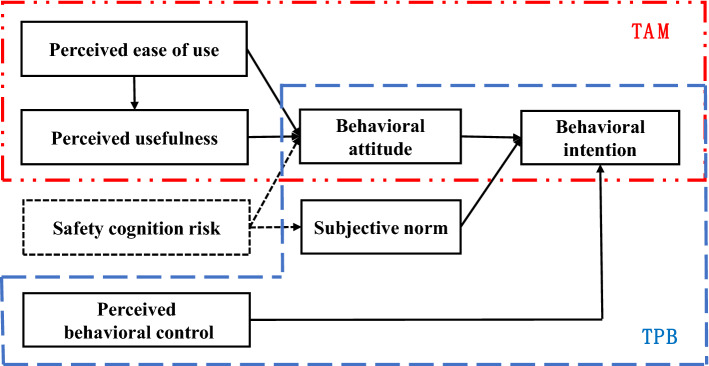
The improved theory of planned behavior and technology acceptance fusion model.

To reasonably explain the changing process of AVs accidents affecting user acceptance of AVs, the study designed an improved model of technology acceptance based on safety cognition risk. The perceived usefulness and perceived ease of use constructs from the original TAM were added to the improved model, constituted the positive factors in user perceptions toward ADT, and served as positive influence paths for behavioral attitude^18^, as detailed in the red box in Fig. 1. Compared with the original TAM model, the evolution process of the TAM2 and TAM3 models suggests that factors at the perceptual level are forward drivers that influence behavioral intention^19^, while existing research models lack latent variables describing user perceptions of external inadequacies or self-inadequacies. Therefore, this study proposed safety cognition risk, which was used to characterize the perceived risk of personal cognitive biases in ADT refracted by AVs accidents in the field of traffic safety, and as a negative influencing factor.

Based on the psychological factors that influence behavioral intention revealed by the TPB (see the blue box in Fig. 1), this study redesigned the paths that portray the influence of perceptual factors of external information on psychological factors. After successive AVs accidents, the dissemination of news reports and self-media information has led to a decline in public perceptions of ADT^20^. Therefore, safety cognition risk may connect external environment and subjective norm, and this path may be the main path through which negative external information influences users’ behavioral intention. The negative environmental information may affect users’ subjective norm and behavioral attitude through safety cognition risk, while positive environmental information influences users’ attitude through perceived usefulness and perceived ease of use. Finally, subjective norm and behavioral attitude further influence behavioral intention.

In summary, the theoretical framework of the improved model proposed in this study is shown in Fig. 1. The model consists of seven constructs which form three layers: perception layer, cognitive layer and decision layer. Perceived usefulness, perceived ease of use, and safety cognition risk belong to the variables in the user perception layer. Behavioral attitude, subjective norm and perceived behavioral control belong to the variables in the user’s cognitive layer. Behavioral intention belongs to the factors in the user decision layer. The proposed model covers the influence paths from the user perception layer to the cognitive layer and then to the decision layer. Further, the positive and negative information of the external environment is perceived through different paths, which enriches the perceptual and cognitive paths of different information and adds to the theoretical study of the psychological activities of users affected by traffic accidents from the perspective of technology acceptance. In addition, seven constructs form eight influence paths whose corresponding hypotheses are as follows.

In the perception layer of the improved model, combining the definitions in TAM and inherent attribute of automated driving, perceived ease of use in this study refers to that AVs have a series of intelligent functions that are easy to operate, as well as a human–machine interface that matches to people’s driving habits^21^. Perceived usefulness refers to the ability of AVs to meet the needs of automated driving, driving safety, smart mobility, and personalization, and is a decisive factor influencing the acceptance of ADT. The influential role of perceived usefulness and perceived ease of use is determined according to TAM^22^. In addition, safety cognition risk is presented as a novel concept in this study, which refers to the perceived risk of AVs accidents that may result from individuals’ cognitive biases of ADT attributes in the field of traffic safety. It has a negative influence effect and may negatively affect both behavioral attitude and subjective norm as described above.

### Hypothesis 1

*Positive effect of perceived ease of use on behavioral attitude.*

### Hypothesis 2

*Positive effect of perceived usefulness on behavioral attitude.*

### Hypothesis 3

*Positive effect of perceived ease of use on perceived usefulness.*

### Hypothesis 4

*Negative effect of safety cognition risk on behavioral attitude.*

### Hypothesis 5

*Negative effect of safety cognition risk on subjective norm.*

Perceived behavioral control is an individual’s perception of how easy or difficult it is to operate and control AVs based on their general qualities and the opportunities and resources they have^23^. Thus, perceived behavioral control measures an individual’s ability to master and control AVs and is a major cognitive factor influencing user acceptance. Behavioral attitudes refer to the positive or negative opinions that individuals hold about the use of AVs^24^. Behavioral intention refers to the willingness to use AVs that is the main influencing factor in determining the occurrence of actual behavior. The relationships between behavioral intention and behavioral attitude, subjective norm, and perceived behavioral control in this study were hypothesized based on the elaboration of TPB.

### Hypothesis 6

*Perceived behavioral control has a positive effect on behavioral intention.*

### Hypothesis 7

*Behavioral attitude has a positive effect on behavioral intention.*

Subjective norm refers to the external pressures that individuals feel about whether or not to use AVs. It is mainly expressed as the opinions of important people or groups and media information that have influence on individuals’ behavioral decisions, and is one of the main influencing factors in studying individual psychological changes. For example, negative opinions of family members, friends and colleagues about AVs, or negative news or media reports about AVs may cause a shift in individual behavioral intention due to individuals’ herd mentality and the habit of following the herd^20,25^. However, as users’ own ability to cognize, analyze and judge information continues to improve, the influence of subjective norm on the acceptance of AVs is mainly manifested in three effects: rejection, neutrality and acceptance.

### Hypothesis 8

*Subjective norm has a positive influence on behavioral intention.*

## Factor analysis and model validation

The experimental protocol of this study was approved by the ethics committee of Chang’an University. All methods reviewed by the ethics committee of Chang’an University were performed in accordance with the Declaration of Helsinki and relevant regulations. We have obtained written informed consent from all study participants.

Meanwhile, the questionnaire was developed specifically based on the potential variables in the improved model and the purpose of this study, and its main part consisted of 7 constructs and 25 question items. A total of 315 questionnaires were distributed online and offline, and 295 questionnaires were returned, with an effective rate of 93.65%. Firstly, the exploratory factor analysis was performed on the collected data to ensure the measurement accuracy of the questionnaire designed in this study. Secondly, the sample data were checked for reliability and validity to test the effectiveness and consistency of the data^26^. Finally, the improved model was verified by path coefficient analysis.

### Exploratory factor analysis

The KMO and Bartlett’s sphericity tests were implemented on the sample data by SPSS version 22 software. The KMO is 0.904 (> 0.8), indicating that the sample data is suitable for performing exploratory factor analysis. In addition, the chi-square value of Bartlett’s sphericity test is 8621.973 (*p* < 0.001), which passes the significance test. It means that the null hypothesis is rejected, further indicating the suitability for exploratory factor analysis.

Exploratory factor analysis was performed by combining the extraction method of principal component analysis and the factor rotation of Kaiser’s maximum variance method. After the completion of the maximum convergence iteration, a total of 7 potential dimensions are extracted with an explanatory power of 67.483%. In addition, 7 potential dimensions are also extracted from the rotated principal component matrix. Except for SCR1 “Risk of distracted driving in automatic driving mode.” with a loading of 0.395, the other factor loadings of SCR and the factor loadings of PU, PEU, BT, PBC, SN, and BI are all greater than the critical value of 0.6^27^.

Therefore, after deleting item SCR1, the results of the re-executed factor analysis are shown in Table 1. A total of 24 items passed the exploratory factor analysis. Moreover, the extracted 7 dimensions can explain 68.221% of the total variance, and the extracted safety cognition risk can explain 8.925% of the total variance. It implies that the sample data can adequately reflect the original data^28^. The results indicate that the seven constructs that constitute the improved model are supported.

**Table 1:** Results of exploratory factor analysis.

Construct	Number of items	Factor loading	Eigenvalue	Variance contribution rate (VCR)	Cumulative VCR
Behavioral intention (BI)	3	0.847	4.384	18.266	18.266
Behavioral attitude (BA)	4	0.814	3.051	12.713	30.978
Subjective norm (SN)	4	0.787	2.477	10.323	41.301
Safety cognition risk (SCR)	3	0.763	2.142	8.925	50.226
Perceived usefulness (PU)	4	0.728	1.716	7.149	57.375
Perceived behavioral control (PBC)	3	0.715	1.538	6.408	63.783
Perceived ease of use (PEOU)	4	0.656	1.065	4.438	68.221

### Reliability and validity assessment

To ensure the internal consistency of the sample data, reliability analysis was performed. The results show that the correlation coefficients between items and the total are all greater than the critical value of 0.5, and the correlation coefficients between the items within the constructs are all greater than the threshold of 0.3. As shown in Table 2, the Cronbach coefficients of the constructs are all larger than the threshold value of 0.7. The test results indicate that the sample data pass the reliability test.

**Table 2:** Assessment of reliability and validity.

Construct	Reliability	Convergent validity	Discriminant validity						
	Cronbach`s α	AVE	SN	PU	BA	PEOU	BI	SCR	PBC
SN	0.881	0.627	0.792						
PU	0.869	0.544	0.397	0.738					
BA	0.905	0.681	0.242	0.597	0.825				
PEOU	0.800	0.513	0.344	0.311	0.420	0.716			
BI	0.905	0.731	0.527	0.340	0.575	0.314	0.855		
SCR	0.888	0.604	0.598	0.373	0.642	0.336	0.459	0.777	
PBC	0.857	0.536	0.260	0.212	0.315	0.574	0.543	0.521	0.732

The validity test results are shown in Table 2. The average variance extracted (AVE) of each construct is greater than 0.5, which indicates that the constructs in the improved model meet the convergent validity criteria, implying that the constructs are well represented. Moreover, the correlations within the constructs are all greater than the critical value of 0.7 (*p* < 0.05), and the correlations between different constructs are all less than the critical value of 0.7 (*p* < 0.05)^29^. Therefore, all the constructs of this study have no collinearity problem and have good discriminant validity. In summary, the sample data pass the reliability and validity tests, indicating that the measurement items can truly reflect the constructs in the improved model, proving that the quality of the sample data meets standard.

### Verification of the improved model

**Figure 2:**
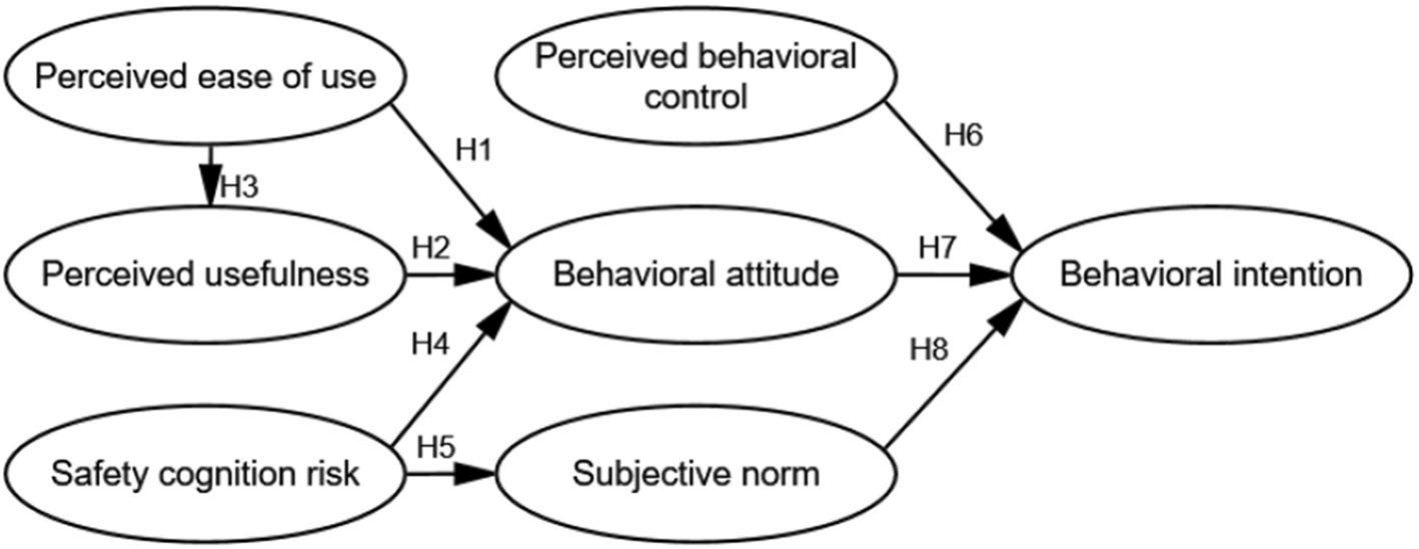
Structural relationship diagram of acceptance model of ADT.

The structural equation model and the research hypothesis relationship of the improved model proposed by the study are shown in Fig. 2. The safety cognition risk is the inverse variable and was done in reverse. To examine the overall fitness of the model, the construct validity analysis of the improved model proposed in this study was conducted by Amos version 24.0.

After removing the options with mismatched MI values and constraining the indices that did not meet the criteria, the obtained model fitting statistics are as follows: the value of Chi/DF is 1.582 (< 3), the Root Mean Square Error of Approximation (RMSEA) is 0.057 (< 0.08), the Goodness of fit index (GFI) is 0. 906 (> 0.9), and the Comparative Fit Index (CFI) is 0.93 (> 0.9)^29,30^. The results show that all the fitting coefficient indicators meet the criteria and the measurement has better structural validity. Therefore, the ADT acceptance model has a good degree of adaptation.

In addition, the results of the normality test of the sample data: the skew coefficient is less than 3, and kurtosis coefficient is less than 8. It indicates that the sample data conform to the normal distribution and can be estimated with maximum likelihood^31^. Furthermore, the path analysis was performed on the improved model proposed in this study by Amos version 24.0, and the fitting results of the path coefficients of the improved model are presented in Table 3.

**Table 3:** Structural equation model and hypothesis testing.

Path	Standardized coefficient β	*p*	Hypotheses finding	
PEOU → BA	0.311	< 0.05	H1	Supported
PU → BA	0.583	< 0.01	H2	Supported
PEOU → PU	0.240	< 0.05	H3	Supported
SCR → BA	0.607	< 0.01	H4	Supported
SCR → SN	0.571	< 0.05	H5	Supported
PBC → BI	0.529	< 0.01	H6	Supported
BA → BI	0.562	< 0.01	H7	Supported
SN → BI	0.428	< 0.01	H8	Supported

The standardized path coefficients between safety cognition risk and behavioral attitude (β = 0.607, *p* < 0.01) and subjective norm (β = 0.571, *p* < 0.05) are significant, indicating support for hypotheses H4 and H5. In addition, the hypotheses between perceived ease of use and perceived usefulness, behavioral attitude, and between perceived usefulness and behavioral attitude are also supported, implying that the research hypotheses H1, H2, and H3 of the improved model hold and are consistent with TAM. Moreover, the hypotheses between behavioral intention and behavioral attitude, subjective norm and perceived behavioral control are also supported, indicating that hypotheses H6, H7, and H8 of this study are supported by TPB. In conclusion, all eight hypotheses proposed in this study are supported and the safety cognition risk of AVs has a significant and negative effect on user acceptance.

Further, Table 3 shows that the safety cognition risk regarding ADT has a strong influence on users’ subjective norm and behavioral attitude and indirectly affects the intention to use AVs through these two mediating variables. On the one hand, it exposes that user cognitive bias about ADT brings certain risks to driving safety and affects user acceptance of AVs. On the other hand, it gives us insights into the subjective norm and behavioral attitude that mediate the safety cognition risk and its negative impact on acceptance.

## Mediated and moderated intervention analysis

**Figure 3:**
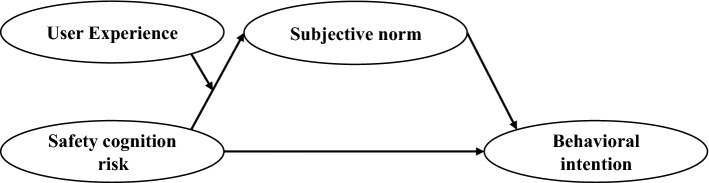
Mediation model with moderating intervention.

From the results of the above model validation, it is revealed that the safety cognition risk regarding ADT has a significant impact on the decrease of user acceptance. To improve user acceptance, this study further explores the mediating and moderating effects of the proposed intervention behaviors on the negative influence relationship between safety cognition risk and user acceptance of automated driving technologies. Based on the improved model proposed and validated in this study, a mediation model with moderating function was developed, as shown in Fig. 3. It is intended to reduce the negative impact of safety cognition risk about ADT on the intention to use AVs, so as to improve user acceptance. The hypotheses supporting the moderated mediation model are as follows.

### H1

*Safety cognition risk is significantly and negatively related to the intention to use AVs.*

### H2

*Subjective norm mediates the relationship between safety cognition risk regarding ADT and intention to use AVs.*

### H3

*User Experience (UX) has a moderating effect on the uplink of the mediated path.*

The moderated mediation model was constructed based on the influence relationship between safety cognition risk and intention to use AVs. In this model, subjective norm, as a mediating variable, was added to analyze its mediating effect on the influence relationship between these two. Meanwhile, UX as a moderator was added in the upstream link of the mediated path for moderating the influence relationship between safety cognition risk and subjective norm.

In the collected literature, it was found that UX researchers and practitioners from academia and industry have difficulty in reaching a consensus on the generic definition, nature and scope of UX, and even show some significant differences^32,33^. The main reason is that most studies conclude that UX is dynamic, fuzzy, context-dependent and subjective in nature. However, the ISO definition of UX refers to a person’s perceptions and responses as a result of using or anticipating the use of a product, system or service^32^. In addition, it has also been suggested that UX focuses on the interaction between a person and something with a user interface^34^.

Based on the above analysis, the UX proposed in this study refers to the modification and enhancement of the user’s perception of the hierarchical classification and anticipated use for the product or technical system in human- machine interaction. The purpose of UX is to improve the anticipated use or the direct negative effects of use and to adjust the relationship between the actual use and the user’s anticipated or subjective imagined use. The UX in this study is concretely expressed as the user’s experience, perception and response to the level of ADT and the automated driving functions it has, as well as the traffic scenarios to which the automated driving functions can be applied.

After popularizing automated driving through a promotional video, the participants had a free test ride experience on L3 level automated driving. During the experience, the safety officer in the driver’s seat only observed the road conditions and did not intervene in the driving when the automated driving system did not send a request to take over. Moreover, the safety officer would introduce the technical level, functions and scenarios to the participants in combination with the driving scenario and human–machine interface. Based on the 295 valid questionnaire data, after verifying the validity and reliability of the data, the mediating effect of subjective norm and the moderating intervention role of UX were analyzed below.

### Common method bias test

The Harman one-way test method was used to test the common method bias for the moderated mediator model^35^. The test results screened a total of 15 factors with eigenvalues greater than 1 and the explained variance of the mediating variable was 14%. Since the explained variance of the mediating variable is less than the 40% threshold, it indicates that common method bias does not exist.

### Correlation matrix of the model

The results of the correlation analysis of the model constructs were obtained by using Amos version 24.0, as shown in Table 4. The correlation coefficient between safety cognition risk regarding ADT and intention to use AVs is − 0.412 with a confidence level of 0.01, indicating a significant negative correlation between these two. Safety cognition risk is significantly negatively correlated with subjective norm (correlation coefficient r = − 0.574, ^**^p < 0.01). However, safety cognition risk is not related to UX. In addition, there are significant positive correlations between UX and subjective norm, and between subjective norm and intention to use AVs. The above results suggest that the hypotheses in the moderated mediation model are supported and safety cognition risk regarding ADT is significantly and negatively correlated with perceived behavioral control.

**Table 4:** Means, standard deviations and correlation matrix of the model constructs.

	Mean	SD	SCR	SN	BI	UX
SCR	5.12	0.85	1			
SN	4.93	0.91	− 0.574**	1		
BI	4.97	1.01	− 0.412**	0.542**	1	
UX	5.25	1.77	− 0.098	0.309**	0.764**	1

### Moderated mediation model test

First, the mediation effect test of SN was performed on the moderated mediation model. Model 4 in the SPSS macro program PROCESS was selected^36^, and educational degree and driving age were set as control variables. The results show that the total effect of safety cognition risk on intention to use AVs is 0.494 (^***^p < 0.001, CI [0.368, 0.620]). The direct effect is 0.179, (^*^p < 0.05, CI [0.039, 0.320]). In addition, the mediating effect of subjective norm is 0.315 (^*^p < 0.05, CI [0.204, 0.438]), Where the effect of safety cognition risk on subjective norm is 0.62 (^***^p < 0.001, CI [0.518,0.722]), and the effect of subjective norm on Behavioral Intention is 0.508 (^***^p < 0.001, CI [0.378,0.637]), accounting for 63.77% of the total effect. It implies that subjective norm has a significant mediating effect and mediates to some extent the negative relationship between safety cognition risk and behavioral intention.

Next, the moderating effect of UX was tested for the moderated mediation model. Model 7 was chosen in the SPSS macro program PROCESS^37^, as well as setting educational degree and driving age as control variables. As shown in Table 5, the results indicate that the product term of safety cognition risk and UX has a significant effect on subjective norm (^**^p < 0.01, CI [0.026, 0.140]). It implies the existence of a significant moderating effect of UX.

**Table 5:** Mediated role with moderation.

Regression equations		Fitting coefficients		Significance of regression coefficients		
Outcome variables	Predictive variables	R^**2**^	F	β	CI	t
SN	SCR	0.411	67.722***	− 0.562***	[− 0.464, − 0.661]	− 11.256
	UX			0.14***	[0.094, 0.186]	5.956
	SCR × UX			0.083**	[0.026, 0.140]	2.872
BI	SN	0.309	65.316***	0.508***	[0.378, 0.637]	7.684
	SCR			− 0.179*	[− 0.039, − 0.320]	− 2.515

Furthermore, the indirect effect of safety cognition risk on behavioral intention was tested when the moderating variable UX was at different levels. After centering the moderating variable UX by the mean center for products method, the UX levels were divided into three levels by using the Mean and Mean ± 1SD method: low level (UX = − 1.772), mean level (UX = 0), and high level (UX = 1.747). The mediating effect value of subjective norm was 0.359 (95% CI [0.235, 0.508]) at the high UX level and 0.211 (95% CI [0.108, 0.348]) at the low UX level. It suggests that UX can moderate the indirect effect of safety cognition risk on intention to use AVs. Moreover, it also means that safety cognition risk regarding ADT has a greater impact on intention to use AVs at low level of UX.

**Figure 4:**
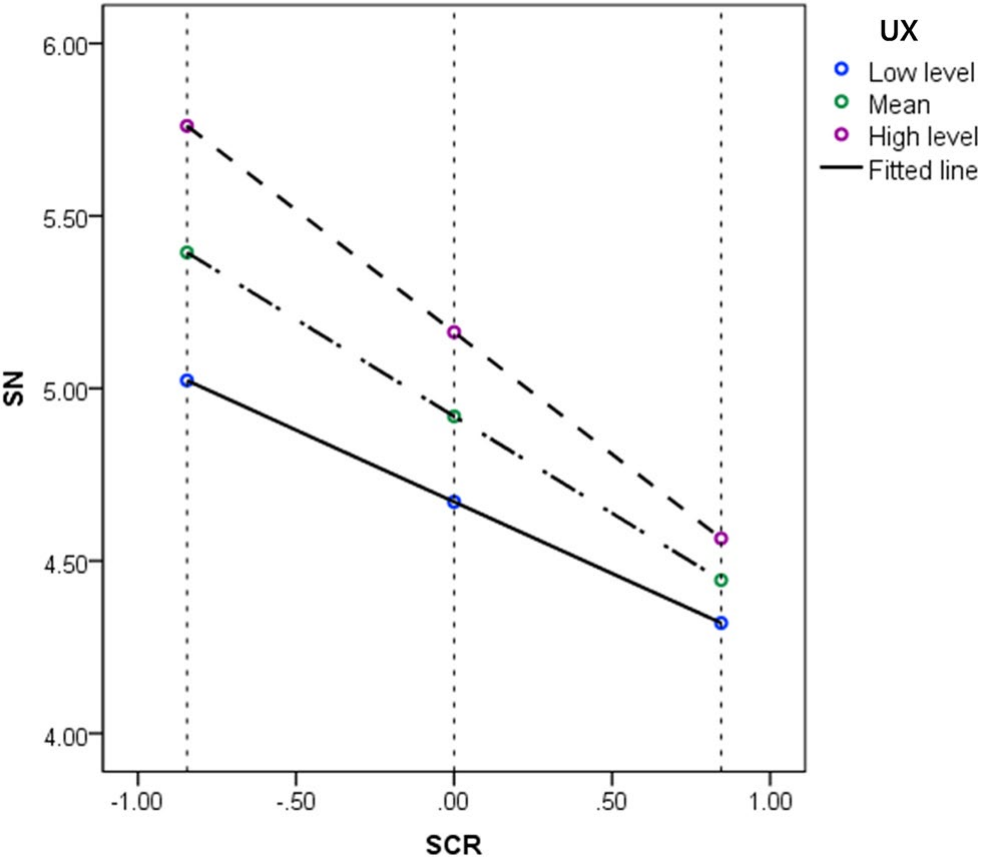
Moderating effect of user experience levels.

In addition, to further analyze the moderating effect of UX, a simple slope test was performed on the high, mean and low levels of UX^38^. The moderating effect of different UX levels on the influence relationship between safety cognition risk and subjective norm is shown in Fig. 4. When UX is at low level, there is a strong negative effect of safety cognition risk on subjective norm (Bsimple = − 0.707, *p* < 0.001, CI [− 0.583, − 0.831]). In contrast, the negative effect of safety cognition risk on subjective norm shows a significant trend of attenuation when UX is at high levels (Bsimple = − 0.415, *p* < 0.001, CI [− 0.260, − 0.570). The results suggest the existence of a moderating effect of UX on the relationship between safety cognition risk and subjective norm.

The negative effect of safety cognition risk on subjective norm is significantly mitigated when the UX is better under the same safety cognition risk. The results indicate that the effect of safety cognition risk on subjective norm is significantly different at the three levels of the moderating variable UX.

## Discussion and conclusion

The study aims to verify the relationship between the safety riskiness of AVs and the decrease in acceptance and to improve the influence relationship between these two. In the improved model proposed by this study, a new factor safety cognition risk was proposed, which was used to characterize the perceived risk of describing personal cognitive biases in ADT refracted by AVs accidents. After the exploratory factor analysis and validation factor analysis of the sample data were passed, the path analysis of the structural equation model verified that all the assumptions in the theoretical model were valid. It is found that safety cognition risk about ADT is significantly and negatively correlated with acceptance. It means that user cognitive bias about ADT brings certain risks to driving safety and affects user acceptance of AVs.

Moreover, the proposed theoretical model is able to explain the process of how safety cognition risk about ADT influences user acceptance. The process is that the risk information of the ADT causes the users to perceive potential risks of using it, and next acts on the behavioral intention through two psychological variables, behavioral attitude and subjective norm, which ultimately causes the change in user acceptance. The finding that safety cognition risk indirectly affects intention to use AVs through subjective norm provides a theoretical basis for the development of strategies to intervene in the negative effects of safety cognition risk.

In addition, this study further explored how to enhance user acceptance by improving the influential relationship between safety cognition risk and acceptance of AVs. A moderated mediation model based on subjective norm was designed, and user experience was added to the model as a moderating variable. The analysis results of the mediation model shows that the mediation effect of subjective norm is significant, and the user experience degree has a significant moderating effect on both the mediation effect of subjective norm and the influence generated by the safety cognition risk about AVs.

This study explains the recent decline in public acceptance of AVs, analyzes the main factors influencing the decline, and also finds the main variables moderating the change in acceptance. It is concluded that behavioral interventions based on user experience and subjective norm have a significant effect in moderating user acceptance of AVs. Findings of this study can provide theoretical support and methodological guidance for improving acceptance of ADT. Future research can look for intervention methods to reduce the impact of risk hazards of ADT on acceptance, starting from psychological activity variables or external environmental factors, so that people can correctly understand ADT and have a good experience, and they are more willing to accept it.

## Data Availability

The datasets generated during the current study are available in the [Figshare] repository, [https://doi.org/10.6084/m9.figshare.19164635].
